# Simultaneous Determination of Potassium Clavulanate and Amoxicillin Trihydrate in Bulk, Pharmaceutical Formulations and in Human Urine Samples by UV Spectrophotometry

**Published:** 2010-12

**Authors:** Rajinder Singh Gujral, Sk Manirul Haque

**Affiliations:** *Vardhman Life Sciences Pvt. Ltd., A–7, Sipcot Industrial Complex, Cuddalore (Tamilnadu), India*

**Keywords:** potassium clavulanate, amoxicillin trihydrate, uv, spectrophotometer, bulk drug, pharmaceutical formulation, human urine samples

## Abstract

A simple and sensitive UV spectrophotometric method was developed and validated for the simultaneous determination of Potassium Clavulanate (PC) and Amoxicillin Trihydrate (AT) in bulk, pharmaceutical formulations and in human urine samples. The method was linear in the range of 0.2–8.5 μg/ml for PC and 6.4–33.6 μg/ml for AT. The absorbance was measured at 205 and 271 nm for PC and AT respectively. The method was validated with respect to accuracy, precision, specificity, ruggedness, robustness, limit of detection and limit of quantitation. This method was used successfully for the quality assessment of four PC and AT drug products and in human urine samples with good precision and accuracy. This is found to be simple, specific, precise, accurate, reproducible and low cost UV Spectrophotometric method.

## INTRODUCTION

**Figure 1:**
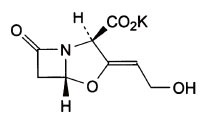
Structure of Potassium Clavulanate.

**Figure 2:**
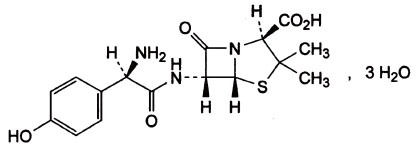
Structure of Amoxiclllin Trihydrate.

Potassium Clavulanate (PC) is chemically known as Potassium (Z)-(2R, 5R)-3-(2-hydroxyethylidene)-7-oxo-4-oxa-1-azabicyclo [3.2.0]-heptene-2-carboxylate [C_8_H_8_KNO_5_, CAS No: 61177-45-5, MW.237.3] (Figure 1). Amoxicillin Trihydrate (AT) is (2S, 5R, 6R)-6-[(R)-(−)-2-Amino-2-(p-hydroxyphenyl) acetamido]-3, 3-dimethyl-7-oxo-4-thia-1-azabicyclo[3.2.0] heptane-2-carboxylic acid trihydrate [C_16_H_19_N_3_O_5_S•3H_2_O, CAS No: 61336-70-7, MW. 419.46] (Figure 2).

Amoxicillin Trihydrate is semisynthethic antibiotic with a broad spectrum of bactericidal activity against many gram-positive and gram-negative microorganisms. AT is, however susceptible to degradation by β-lactamases, and therefore, the spectrum of activity does not include organisms which produce these enzymes. Clavulanic acid is a β-lactam, structurally related to the penicillins, which possesses the ability to inactivate a wide range of β-lactamase enzymes commonly found in microorganisms resistant to penicillins and cephalosporins.

PC and AT are well absorbed from the gastrointestinal tract after oral administration of amoxicillin and potassium clavulanate. Dosing in the fasted or fed state has minimal effect on the pharmacokinetics of amoxicillin. While PC and AT can be given without regard to meals, absorption of clavulanate potassium when taken with food is greater relative to the fasted state. The relative bioavailability of clavulanate was reduced when PC and AT was dosed at 30 and 150 minutes after the start of high-fat breakfast. The safety and efficacy of PC and AT have been established in clinical trials where PC and AT was taken without regard to meals. The use of potassium clavulanate with penicillins has been associated with an increased incidence of cholestatic jaundice and acute hepatitis during therapy, particularly in men and those aged over 65 years.

The official methods (1, 2) were developed for the determination of PC and AT individually in its pure form but there are some analytical methods (3-7) developed for the quantitation of amoxicillin-clavulanate dosage forms.

This paper reports a simple, sensitive and accurate Spectrophotometric method for the simultaneous determination of potassium clavulanate and amoxicillin trihydrate. This method is based on the direct measurement of native absorbance of the drug at 205 and 271 nm against the reagent blank respectively for potassium clavulanate and amoxicillin trihydrate.

## EXPERIMENTAL

### Apparatus

Spectral runs were made on UV 3000^+^ UV/VIS spectrophotometer (LABINDIA^®^, Mumbai, India) with 1 cm matched glass cell.

### Materials and Reagents

- Potassium Clavulanate (Vardhman Life Sciences Pvt. Ltd, Tamilnadu, India) was used as working standard.
- Amoxicillin Trihydrate (Vardhman Chemtech Ltd, Punjab, India) was used as working Standard.
- Pharmaceutical formulations of such as Augmentin 625 (GlaxoSmithKline, Mumbai, India), Flemiclav 625 (Vapi Care Pharma Pvt. Ltd, Solan, India), Moxikind-CV 625 (Mankind Pharma Ltd, New Delhi, India) and Clavam 625 (Alkem Laboratories Ltd, Mumbai, India) were purchased from local markets.
- Sodium carbonate was purchased from Qualigens fine chemicals (Mumbai, India).
- Sodium bicarbonate was purchased from Qualigens fine chemicals (Mumbai, India).
- Urine samples were obtained from healthy volunteers.
- Carbonate buffer of pH 9.4 was prepared by dissolving 26.5 gm sodium carbonate and 21.0 gm sodium bicarbonate in 500 ml distilled water.

### Determination of appropriate UV wavelength

A suitable wavelength was required for simultaneous determination of PC and AT. The appropriate wavelength for the determination of PC and AT was determined by wavelength scanning over the range 190–500 nm with a UV 3000^+^ UV/VIS spectrophotometer (LABINDIA^®^, Mumbai, India).

### Standard PC and AT Solution

A stock solution of PC (50 μg/ml) and AT (100 μ/ml) was prepared by dissolving 5 and 10 mg PC and AT respectively in 100 ml volumetric flasks with double distilled water. The stock solutions were used to prepare the working solutions by suitable dilutions with distilled water. The solutions were stable at least 10 days in room temperature.

## METHODS

### Procedure for the determination of PC and AT

Aliquots of stock solution 50 μg/ml and 100 μg/ml were transferred into a set of 50 ml volumetric flasks and volumes were completed to the mark with distilled water to produce solutions in the concentration range 0.2–8.5 and 6.4–33.6 μg/ml for PC and AT. Absorbance was measured at 205 and 271 nm respectively for PC and AT against the reagent blank. Calibration graphs were constructed by plotting absorbance against the final concentration of PC and AT.

### Procedure for determination of PC and AT in pharmaceutical formulations

One tablet (claiming 625 mg) was accurately weighed and finely powdered. A quantity of the powder equivalent to 125 mg of PC and 500 mg AT was extracted by shaking with 50 ml of distilled water, followed by another two extractions each with 50 ml distilled water. After passing through a 0.45 μm Millipore filter, the solution was diluted with distilled water to obtain a concentration of about 1250 and 5000 μg/ml respectively for PC and AT. It was further diluted according to the need and then analyzed following the proposed procedures. The nominal content of the tablet was calculated either from the previously plotted calibration graphs or using regression equation.

### Procedure for determination of PC and AT in human urine samples

Aliquot volumes of human urine samples were transferred into small separating funnel. 20 ml of carbonate buffer pH9.4 was added and solution was mixed well. The solution was then extracted with 3 × 20 ml diethyl ether. The ether extract was collected and evaporated. The residue was dissolved in 10 ml distilled water and above general procedure was then followed. The amount of PC and AT was obtained from the calibration graphs or corresponding regression equation.

## METHOD VALIDATION

The method was validated for selectivity, linearity, precision, accuracy, recovery and stability according to the principles of the Food and Drug Administration (FDA) industry guidance (8). Validation of analytical procedures is a vital aspect not just for regulatory purposes, but also for their efficient and reliable long–term application. The ICH guidelines achieved a great deal in harmonizing the definitions of required validation parameters, their calculation and interpretation. It is the responsibility of the analyst to identify parameters which are relevant to the performance of given analytical procedure as well as to design proper validation protocols including acceptance criteria and to perform an appropriate evaluation. The International Conference on the Harmonization of the Technical Requirements for Registration of Pharmaceuticals for Human Use has harmonized the requirements in two guidelines (9, 10). The first one summarizes and defines the validation characteristics needed for various types of test procedures, the second one extends the previous test to include the experimental data required and some statistical interpretation. These guidelines serve as a basis worldwide both for regulatory authorities and industry and bring the importance of a proper validation to the attention of all those involved in the process of submission. Nowadays, the validation characteristics needed for the various test procedures and their general requirements are well understood. The essential question to be answered is on the suitability of the calibration mode to be used in the test procedure. It should be noted that in most cases only a qualitative statement is needed.

The stability of the working sample solutions at room temperature was evaluated with the help of UV spectra. The specificity and selectivity of the proposed method was evaluated by estimating the amount of PC and AT in the presence of common excipients colloidal silicon dioxide, magnesium stearate, microcrystalline cellulose, polydextrose, titanium dioxide.

The linearity of the proposed method was constructed for PC and AT reference standard solution by plotting concentration of the compound versus the absorbance. The linearity was evaluated by linear regression analysis, which was calculated by the least square regression method. The parameters LOD and LOQ were determined on the basis of response and slope of the regression equation. The accuracy and precision of the method was evaluated within the linear range based on the analysis of PC and AT reference standard samples and pharmaceutical formulations at 2.0, 5.0, 8.0 and 8.0, 20.0 and 32.0 μg/ml. Five independent analysis were performed at each concentrations level within one day (intraday precision) as well as for five consecutive days (interday precision). The accuracy was ascertained by recovery studies using the standard addition method. The proposed method was used for estimation of PC and AT from tablet after spiking with 50, 150, 250 % and 100, 300, 500 % of additional pure drug. The amount of PC and AT was determined from the regression equation.

## RESULTS AND DISCUSSIONS

The absorbance-concentration plot for the proposed method was found to be rectilinear over the range of 0.2–8.5 and 6.4–33.6 μg/ml respectively for PC and AT. Linear regression analysis of calibration data gave the regression equation cited in Table 1 with correlation coefficients close to unity.

**Table 1:** Summary of optical and regression characteristics of the proposed method

Parameters	Potassium Clavulanate	Amoxicillin Trihydrate

Linear dynamic range (μg/ml)	0.2–8.5	6.4–33.6
Regression equation^a^	Y=0.0575X–0.003	Y=0.0174X–0.0254
Slope	0.0575	0.0174
Intercept	0.003	0.0254
Correlation coefficient (r)	0.9984	0.9964

a With respect to Y = a + b *X*, where X is the concentration in μg/ml, Y is Absorbance.

The within day precision assays were carried out through replicate analysis (n=5) of PC and AT corresponding to 2.0, 5.0, 8.0 and 8.0, 20.0, 32.0 μg/ml respectively for PC and AT. The interday precision was evaluated through replicate analysis of the pure drug samples for five consecutive days at the same concentration levels as used in within day precision. The results of these assays are reported in Table 2. As can be seen from Table 2 that the recovery and RSD values for within day precision were in the range of 99.826–99.957%, 99.856–100.036% and 0.358–0.954%, 0.098–0.394% respectively for PC and AT; recovery and RSD values for interday precision were in the range of 99.757–100.174%, 99.785–99.828 % and 0.424–1.132%, 0.353–0.644% respectively for PC and AT. The precision results are satisfactory. The intraday and interday precision assays were also carried for PC and AT in pharmaceutical formulations. The results are summarized in Table 3 and 4. As can be seen from Table 3 that the recovery and RSD values for intraday precision were in the ranges 99.269–99.980%; 0.420–1.326%, recovery and RSD values for interday precision were in the ranges 98.783–99.826% ; 0.512–1.566 % for Potassium Clavulanate.

**Table 2:** Summary of accuracy and precision results of the proposed method in pure form

Proposed methods	Amount (μg/ml)		RSD	REC.	SAE b	C.L.^c^
	Taken	Found ± SD^a^				

Potassium Clavulanate						
Intraday assay	2.00	1.997 ± 0.019	0.954	99.826	8.5 × 10^−3^	2.4 × 10^−2^
	5.00	4.991 ± 0.028	0.551	99.826	1.2 × 10^−2^	3.4 × 10^−2^
	8.00	7.997 ± 0.029	0.358	99.957	1.3 × 10^−2^	3.6 × 10^−2^
Interday assay	2.00	2.004 ± 0.023	1.132	100.174	1.0 × 10^−2^	2.8 × 10^−2^
	5.00	4.988 ± 0.024	0.671	99.757	1.5 × 10^−2^	4.2 × 10^−2^
	8.00	7.993 ± 0.034	0.424	99.913	1.5 × 10^−2^	4.2 × 10^−2^
Amoxicillin Trihydrate						
Intraday assay	8.00	7.989 ± 0.032	0.394	99.856	1.4 × 10^−2^	3.9 × 10^−2^
	20.00	19.989 ± 0.031	0.158	99.943	1.4 × 10^−2^	3.9 × 10^−2^
	32.00	32.012 ± 0.032	0.098	100.036	1.4 × 10^−2^	3.9 × 10^−2^
Inter day assay	8.00	7.977 ± 0.051	0.644	99.713	2.3 × 10^−2^	6.4 × 10^−2^
	20.00	19.965 ± 0.070	0.353	99.828	3.1 × 10^−2^	8.7 × 10^−2^
	32.00	31.931 ± 0.125	0.390	99.785	5.6 × 10^−2^	1.6 × 10^−1^

Mean for 5 independent analyses.

a SD, standard deviation, RSD, relative standard deviation;

b SAE, standard analytical error;

c C.L., confidence limit at 95 % confidence level and 4 degrees of freedom (t=2.776).

**Table 3:** Summary of accuracy and precision results of the proposed method for Potassium Clavulanate pharmaceutical formulations

Proposed methods	Amount (μg/ml)		RSD	REC.	SAE b	C.L. c
	Taken	Found ± SD a				

Intra day assay						
Augmentin – 625	2.00	1.986 ± 0.019	0.959	99.304	0.0085	0.0237
Augmentin – 625	5.00	4.974 ± 0.035	0.699	99.478	0.0156	0.0432
Augmentin – 625	8.00	7.986 ± 0.042	0.520	99.826	0.0186	0.0515
Flemiclav – 625	2.00	1.990 ± 0.016	0.782	99.980	0.0070	0.0193
Flemiclav – 625	5.00	4.970 ± 0.029	0.575	99.409	0.0128	0.0355
Flemiclav –625	8.00	7.983 ± 0.048	0.597	99.783	0.0213	0.0591
Moxikind – CV 625	2.00	1.993 ± 0.023	1.171	99.652	0.0104	0.0290
Moxikind – CV 625	5.00	4.967 ± 0.026	0.531	99.339	0.0118	0.0327
Moxikind – CV 625	8.00	7.979 ± 0.045	0.564	99.739	0.0201	0.0559
Clavam – 625	2.00	1.989 ± 0.027	1.326	99.478	0.0118	0.0327
Clavam – 625	5.00	4.963 ± 0.026	0.531	99.269	0.0118	0.0327
Clavam – 625	8.00	7.969 ± 0.033	0.420	99.609	0.0150	0.0415
Inter day assay						
Augmentin – 625	2.00	1.997 ± 0.023	1.136	99.826	0.0101	0.0281
Augmentin – 625	5.00	4.964 ± 0.036	0.727	99.270	0.0161	0.0448
Augmentin – 625	8.00	7.979 ± 0.048	0.605	99.739	0.0216	0.0599
Flemiclav – 625	2.00	1.983 ± 0.025	1.241	99.130	0.0101	0.0305
Flemiclav – 625	5.00	4.967 ± 0.034	0.683	99.339	0.0152	0.0421
Flemiclav – 625	8.00	7.976 ± 0.061	0.765	99.695	0.0273	0.0757
Moxikind – CV 625	2.00	1.986 ± 0.031	1.566	99.304	0.0139	0.0386
Moxikind – CV 625	5.00	4.960 ± 0.038	0.760	99.200	0.0169	0.0468
Moxikind – CV 625	8.00	7.972 ± 0.059	0.733	99.652	0.0262	0.0726
Clavam – 625	2.00	1.986 ± 0.019	0.959	99.304	0.0085	0.0237
Clavam – 625	5.00	4.939 ± 0.030	0.610	98.783	0.0135	0.0374
Clavam – 625	8.00	7.965 ± 0.041	0.512	99.565	0.0182	0.0506

a Mean for 5 independent analyses; RSD, relative standard deviation;

b SAE, standard analytical error;

c C.L., confidence limit at 95 % confidence level and 4 degrees of freedom (t=2.776).

As can be seen from Table 4 that the recovery and RSD values for intraday precision were in the ranges 99.707–99.820%; 0.327–0.891%, recovery and RSD values for interday precision were in the ranges 98.994–99.713%; 0.424–1.101% for Amoxicillin Trihydrate.

**Table 4:** Summary of accuracy and precision results of the proposed method for Amoxicillin Trihydrate pharmaceutical formulations

Proposed methods	Amount (μg/ml)		RSD	REC.	SAE b	C.L. c
	Taken	Found ± SD a				

Intra day assay						
Augmentin – 625	8.00	7.943 ± 0.048	0.605	99.282	0.0215	0.0597
Augmentin – 625	20.00	19.943 ± 0.087	0.437	99.713	0.0390	0.1082
Augmentin – 625	32.00	31.943 ± 0.104	0.327	99.820	0.0467	0.1296
Flemiclav – 625	8.00	7.966 ± 0.048	0.604	99.569	0.0215	0.0597
Flemiclav – 625	20.00	19.920 ± 0.094	0.474	99.598	0.0422	0.1172
Flemiclav – 625	32.00	31.920 ± 0.135	0.422	99.749	0.0603	0.1673
Moxikind – CV 625	8.00	7.908 ± 0.048	0.608	98.851	0.0215	0.0597
Moxikind – CV 625	20.00	19.908 ± 0.115	0.577	99.540	0.0514	0.1427
Moxikind – CV 625	32.00	31.874 ± 0.160	0.500	99.605	0.0713	0.1979
Clavam – 625	8.00	7.897 ± 0.070	0.891	98.707	0.0315	0.0874
Clavam – 625	20.00	19.874 ± 0.096	0.484	99.368	0.0430	0.1194
Clavam – 625	32.00	31.828 ± 0.210	0.659	99.461	0.0937	0.2602
Inter day assay						
Augmentin – 625	8.00	7.931 ± 0.065	0.826	99.138	0.0293	0.0814
Augmentin – 625	20.00	19.908 ± 0.091	0.457	99.540	0.0406	0.1128
Augmentin – 625	32.00	31.908 ± 0.154	0.483	99.713	0.0689	0.1915
Flemiclav – 625	8.00	7.954 ± 0.058	0.723	99.425	0.0257	0.0713
Flemiclav – 625	20.00	19.851 ± 0.115	0.579	99.253	0.0514	0.1427
Flemiclav – 625	32.00	31.851 ± 0.170	0.532	99.533	0.0758	0.2104
Moxikind – CV 625	8.00	7.919 ± 0.066	0.827	98.994	0.0293	0.0814
Moxikind – CV 625	20.00	19.851 ± 0.147	0.738	99.253	0.0655	0.0819
Moxikind – CV 625	32.00	31.874 ± 0.179	0.562	99.605	0.0801	0.2222
Clavam – 625	8.00	7.919 ± 0.087	1.101	98.994	0.0390	0.1082
Clavam – 625	20.00	19.839 ± 0.131	0.661	99.195	0.0586	0.0603
Clavam – 625	32.00	31.805 ± 0.135	0.424	99.389	0.0603	0.1673

a Mean for 5 independent analyses; SD, standard deviation; RSD, relative standard deviation;

b SAE, standard analytical error;

c C.L., confidence limit at 95 % confidence level and 4 degrees of freedom (t=2.776).

The proposed method was used for estimating of PC and AT from tablet after spiking with 50, 150, 250 and 100, 300, 500% of additional pure drug respectively. The results are reported in Table 5. As can be seen from Table 5 that the recovery and RSD values were in the ranges 98.634–99.942%, 0.563–1.120% and 98.966–99.885%, 0.303–1.085% respectively. The selectivity of the propose method was ascertained by analyzing standard PC and AT in the presence tablet such as colloidal silicon dioxide, magnesium stearate, microcrystalline cellulose, polydextrose, titanium dioxide. It was observed that the excipients did not interfere with the proposed method.

**Table 5:** Summary of data for the determination of PC and AT in pharmaceutical preparations by standard addition method

Proposed methods	Amount (μg/ml)			Recovery (%)	RSD (%)	SAE b
	Taken	Added	Found ± SD a			

Augmentin 625	2.00	1.00	2.998 ± 0.020	99.942	0.661	0.0089
	2.00	3.00	4.970 ± 0.040	99.409	0.798	0.0177
	2.00	5.00	6.991 ± 0.055	99.878	0.787	0.0245
Flemiclav 625	2.00	1.00	2.991 ± 0.033	99.710	1.088	0.0146
	2.00	3.00	4.964 ± 0.052	99.269	1.039	0.0231
	2.00	5.00	6.915 ± 0.061	98.783	0.882	0.0757
Moxikind CV 625	2.00	1.00	2.995 ± 0.026	99.826	0.861	0.0115
	2.00	3.00	4.974 ± 0.043	99.478	0.856	0.0191
	2.00	5.00	6.928 ± 0.063	98.981	0.915	0.0284
Clavam 625	2.00	1.00	2.988 ± 0.033	99.594	1.120	0.0150
	2.00	3.00	4.960 ± 0.040	99.200	0.799	0.0177
	2.00	5.00	6.904 ± 0.039	98.634	0.563	0.0174
Amoxicillin Trihydrate						
Augmentin 625	5.00	5.00	9.954 ± 0.075	99.540	0.753	0.0335
	5.00	15.00	19.897 ± 0.110	99.483	0.556	0.0494
	5.00	25.00	29.966 ± 0.091	99.885	0.303	0.0406
Flemiclav 625	5.00	5.00	9.943 ± 0.087	99.425	0.877	0.0390
	5.00	15.00	19.862 ± 0.125	99.310	0.627	0.0557
	5.00	25.00	29.954 ± 0.111	99.847	0.369	0.0494
Moxikind CV 625	5.00	5.00	9.908 ± 0.108	99.080	1.085	0.0481
	5.00	15.00	19.839 ± 0.094	99.195	0.476	0.0422
	5.00	25.00	29.943 ± 0.132	99.808	0.442	0.0592
Clavam 625	5.00	5.00	9.896 ± 0.075	98.966	0.757	0.0335
	5.00	15.00	19.816 ± 0.066	99.081	0.331	0.0293
	5.00	25.00	29.920 ± 0.137	99.732	0.459	0.0614

a Mean for 5 independent analyses;

b SAE, standard analytical error; RSD, relative standard deviation.

The performance of the proposed method was studied with other existing HPLC method (5). In this case, the Standard Deviation (SD) and Relative Standard Deviation (RSD) values of the proposed method is much better as compared to reported method (Table 6) and the method is not applied for invitro determination of PC and AT in human urine samples. The method is also applied to measure the concentration of other antibiotics such as ticarcilin.

**Table 6:** Assay results of PC and AT in commercial tablet using the proposed and reference method (5)

Method	Recovery (%)	SD (%)	RSD (%)

Potassium Clavulanate			
Proposed	99.61	0.033	0.007
Reference	103.04	2.12	0.412
Amoxycillin Trihydrate			
Proposed	99.82	0.104	0.0008
Reference	102.58	1.18	0.920

SD, standard deviation; RSD, relative standard deviation.

Approximately 50 % to 70 % of the amoxicillin and approximately 25% to 40% of the clavulanic acid are excreted unchanged in urine during the first 6 hours after administration of a single amoxicillin and clavulanate potassium tablet 500 mg/125 mg. The results are accurate for healthy controls (Table 7). The usual adult dose is one 500 mg tablet of amoxicillin/clavulanate potassium every 12 hours or one 250 mg tablet of amoxicillin/clavulanate potassium every 8 hours. For more severe infections and infections of the respiratory tract, the dose should be one 875 mg tablet of amoxicillin/clavulanate potassium every 12 hours or one 500 mg tablet of amoxicillin/clavulanate potassium every 8 hours.

**Table 7:** Application of the proposed UV method to the determination of PC and AT in human urine samples

Amount added (μg/ml)	Amount found (μg/ml)	Recovery (%)

Potassium Clavulanate		
1.00	0.9624	96.240
2.00	1.9581	97.905
3.00	2.9541	98.470
4.00	3.9701	99.253
5.00	4.9860	99.720
6.00	5.9512	99.187
7.00	6.9874	99.820
8.00	7.9745	99.681
X		98.785
RSD		1.239
Amoxicillin Trihydrate		
9.00	8.7841	97.601
12.00	11.7810	98.175
15.00	14.8125	98.750
18.00	17.8712	99.284
21.00	20.8914	99.483
24.00	23.9062	99.609
27.00	26.8950	99.611
30.00	29.8812	99.604
X		99.015
RSD		0.775

RSD, relative standard deviation.

Patients with impaired renal function do not generally require a reduction in dose unless the impairment is severe. Severely impaired patients with a glomerular filtration rate of <30 mL/min. should not receive the 875 mg tablet. Patients with a glomerular filtration rate of 10 to 30 mL/min. should receive 500 mg or 250 mg every 12 hours, depending on the severity of the infection. Patients with a less than 10 mL/min. glomerular filtration rate should receive 500 mg or 250 mg every 24 hours, depending on severity of the infection.

Hemodialysis patients should receive 500 mg or 250 mg every 24 hours, depending on severity of the infection. They should receive an additional dose both during and at the end of dialysis. Hepatically impaired patients should be dosed with caution and hepatic function monitored at regular intervals. Urine pH is used to classify urine as either a dilute acid or base solution. Seven is the point of neutrality on the pH scale. The lower the pH, the greater the acidity of a solution; the higher the pH, the greater the alkalinity. The glomerular filtrate of blood is usually acidified by the kidneys from a pH of approximately 7.4 to a pH of about 6 in the urine. Depending on the person’s acid-base status, the pH of urine may range from 4.5 to 8. The kidneys maintain normal acid-base balance primarily through the reabsorption of sodium and the tubular secretion of hydrogen and ammonium ions. Urine becomes increasingly acidic as the amount of sodium and excess acid retained by the body increases. Alkaline urine, usually containing bicarbonate-carbonic acid buffer, is normally excreted when there is an excess of base or alkali in the body. Secretion of an acid or alkaline urine by the kidneys is one of the most important mechanisms the body uses to maintain a constant body pH.

The formation of renal stones is related to the urine pH. Patients being treated for renal calculi are frequently given diets or medications to change the pH of the urine so that kidney stones will not form. Calcium phosphate, calcium carbonate, and magnesium phosphate stones develop in alkaline urine; when this occurs, the urine is kept acidic. Uric acid, cystine, and calcium oxalate stones precipitate in acidic urine; in this situation, the urine should be kept alkaline or less acidic than normal. This method is suitable for determination PC and AT in urine samples with different pH (8 μg/ml) and having foreign bodies likes crystals and stones. The results (Table 8 & 9) are satisfactorily accurate and precise.

**Table 8:** Application of the proposed method to the determination of PC and AT in urine samples with different pH

pH	Amount found (μg/ml)	Recovery (%)	RSD (%)

Potassium Clavulanate			
4.50	7.510	93.88	2.05
5.00	7.546	94.33	1.89
5.50	7.559	94.49	1.79
6.00	7.690	96.13	1.70
7.00	7.792	97.40	1.60
7.5	7.531	94.12	1.97
8.00	7.068	88.35	2.11
Amoxicillin Trihydrate			
4.50	7.470	92.10	1.99
5.50	7.516	93.25	1.78
6.00	7.539	94.10	1.65
6.50	7.621	95.03	1.58
7.00	7.812	98.40	1.50
7.50	7.561	93.12	1.80
8.00	7.552	94.02	1.90

RSD, relative standard deviation

**Table 9:** Application of the proposed method to the determination of PC and AT in human urine samples with the presence of foreign bodies

Amount added (μg/ml)	Amount found (μg/ml)	Recovery (%)

Potassium Clavulanate		
1.00	0.9420	94.20
2.00	1.9181	95.91
3.00	2.8841	96.14
4.00	3.9201	98.00
5.00	4.9060	98.12
X		96.47
RSD		1.690
Amoxicillin Trihydrate		
8.00	7.7283	96.60
10.00	9.7810	97.81
12.00	11.8720	98.93
14.00	13.4221	95.87
16.00	15.4702	95.69
X		96.98
RSD		1.41

RSD, relative standard deviation.

## CONCLUSIONS

The proposed method does not require any laborious clean up procedure before measurement. In addition, the method has wider linear dynamic range with good accuracy and precision. The method shows no interference from the common excipients and additives. This method is simple, cost affective, fast and efficient method. This may help in analyzing affectivity of this drug in human beings during treatment. Therefore, it is concluded that the proposed method is simple, sensitive and rapid for the determination of potassium clavulanate and amoxicillin trihydrate in bulk, pharmaceutical formulations and in human urine samples.
